# The Pathways of the iRFP713 Unfolding Induced by Different Denaturants

**DOI:** 10.3390/ijms19092776

**Published:** 2018-09-15

**Authors:** Olesya V. Stepanenko, Olga V. Stepanenko, Irina M. Kuznetsova, Konstantin K. Turoverov

**Affiliations:** 1 Laboratory of Structural dynamics, Stability and Folding of Proteins, Institute of Cytology, Russian Academy of Sciences, 4 Tikhoretsky ave, St. Petersburg 194064, Russia; lvs@incras.ru (O.V.S.); sov@incras.ru (O.V.S.); imk@incras.ru (I.M.K.); 2 Department of Biophysics, Peter the Great St. Petersburg Polytechnic University, Polytechnicheskaya str., 29, St. Petersburg 194064, Russia

**Keywords:** iRFP713, protein unfolding, protein aggregation, intermediate state, guanidinium cation

## Abstract

Near-infrared fluorescent proteins (NIR FPs) based on the complexes of bacterial phytochromes with their natural biliverdin chromophore are widely used as genetically encoded optical probes for visualization of cellular processes and deep-tissue imaging of cells and organs in living animals. In this work, we show that the steady-state and kinetic dependencies of the various spectral characteristics of iRFP713, developed from the bacterial phytochrome *Rp*BphP2 and recorded at protein unfolding induced by guanidine hydrochloride (GdnHCl), guanidine thiocyanate (GTC), and urea, differ substantially. A study of the unfolding of three single-tryptophan mutant forms of iRFP713 expectedly revealed that protein unfolding begins with the dissociation of the native dimer, while the monomers remain compact. A further increase in the denaturant concentration leads to the formation of an intermediate state of iRFP713 having hydrophobic areas exposed on the protein surface (I). The total surface charge of iRFP713 (pI 5.86) changes from negative to positive with an increase in the concentration of GdnHCl and GTC because the negative charge of glutamic and aspartic acids is neutralized by forming salt bridges between the carboxyl groups and GdnH^+^ ions and because the guanidinium cations bind to amide groups of glutamines and asparagines. The coincidence of both the concentration of the denaturants at which the intermediate state of iRFP713 accumulates and the concentration of GdnH^+^ ions at which the neutralization of the surface charge of the protein in this state is ensured results in strong protein aggregation. This is evidently realized by iRFP713 unfolding by GTC. At the unfolding of the protein by GdnHCl, an intermediate state is populated at higher denaturant concentrations and a strong aggregation is not observed. As expected, protein aggregates are not formed in the presence of the urea. The aggregation of the protein upon neutralization of the charge on the macromolecule surface is the main indicator of the intermediate state of protein. The unfolded state of iRFP713, whose formation is accompanied by a significant decrease in the parameter *A*, was found to have a different residual structure in the denaturants used.

## 1. Introduction

Near-infrared fluorescent proteins (NIR FPs) are widely used optical probes for real-time visualization of molecular processes, from the level of the single cell to that of the whole organism with a high resolution [1,2,3,4]. The possibility of manipulating NIR FPs derived from bacterial phytochromes (BphPs) as easily as genetically encoded probes arises from their ability to bind biliverdin IXα (BV) [5], which serves as the chromophore in these proteins and is produced in mammalian cells in sufficient amounts as a result of heme catabolism [6].

The previous study of unfolding dimeric and monomeric NIR FPs yielded data essential for understanding the formation of the spectral properties of NIR FPs. It has been shown that the alternative covalent binding of the BV chromophore to the cysteine residue in the Per-ARNT-Sim repeats (PAS) domain or to the cysteine residue introduced into the conservative -SPXH- motif of the cGMP phosphodiesterase/adenylate cyclase/FhlA transcriptional activator (GAF) domain affects the spectral properties of NIR FPs [7,8] and their stability [9,10]. Thus, the BV attachment to the Cys residue in the GAF domain results in a 30–40 nm blue shift of absorbance and fluorescence of NIR FPs compared to those of NIR FPs bearing the conserved Cys in the PAS domain. The interaction of the BV chromophore with dimeric NIR FPs is regulated by allosteric influence of the monomers on each other in the protein, to which spectral characteristics of NIR FPs are sensitive [7,10].

Comparative studies of the denaturation and renaturation processes of iRFP713, engineered from bacterial phytochrome *Rp*BphP2 [11], in the holoform (bound with BV) and the apoform (free of the chromophore) induced by guanidine hydrochloride (GdnHCl) showed that covalently bound to iRFP713 chromophore prevents the refolding of the protein in in vitro experiments [12]. The polypeptide chain of NIR FPs contains an intricate structural element that is rather rare in the proteins [13]—a figure-of-eight knot in which formation both PAS and GAF domains are involved [14]. A range of fundamental study addressed the biological significance of knots in the proteins [15,16,17,18,19,20,21,22,23]. However, the presence of a knot in the structure of iRFP713 does not prevent its effective refolding [12]. It is supposed that the knot is preserved in the chemically denatured state of iRFP713, as was observed for a number of other knotted proteins [24,25]. 

The possibility to test the structural changes in individual domains of iRFP713 by changes in the fluorescence parameters of BV and three tryptophan residues localized in different parts of the macromolecule: one of the tryptophan residues is situated in the dimer interface of the protein; two others are in different protein domains (Supplementary Figure S1), which makes iRFP713 an interesting object for studying protein unfolding processes. Here, we studied the unfolding of iRFP713 in its apo- and holoform states induced by different chemical denaturants. Ionic denaturants GdnHCl and guanidine thiocyanate (GTC) are able to interact with proteins in native and intermediate states [26,27,28,29,30,31,32,33]. In addition to GdnHCl and GTC, we used weak non-ionic denaturant urea. We studied the unfolding of single-tryptophan variants of iRFP713, in which all tryptophan residues except for one have been replaced by phenylalanine. We combined the steady-state and kinetics measurements of changes in different spectral characteristics of iRFP713 during its unfolding. Previously based on the results of kinetic experiments, the scheme of actin unfolding was completely revised [34]. This comprehensive approach allowed us to elucidate the reasons for the significant difference of steady-state and kinetic dependencies of the spectral characteristics of iRFP713 measured at protein unfolding induced by different denaturants. 

## 2. Results and Discussion

### 2.1. The Unfolding of iRFP713 Is Accompanied by the Protein Aggregation

In this work, we studied the denaturation processes of iRFP713 in its apo- and holoform induced by chemical denaturant GTC, GdnHCl, and urea. Conformational changes in the protein in the presence of denaturant were probed by the characteristics of tryptophan fluorescence (fluorescence intensity at registration wavelengths of 320 and 365 nm, parameter *A* and fluorescence anisotropy), spectral characteristics of the chromophore (optical density at the maximum of the far-red absorption band and chromophore fluorescence intensity), light scattering at wavelengths of 295 nm, and ellipticity in the far-ultraviolet (UV) region of the spectrum. 

**Figure 1:**
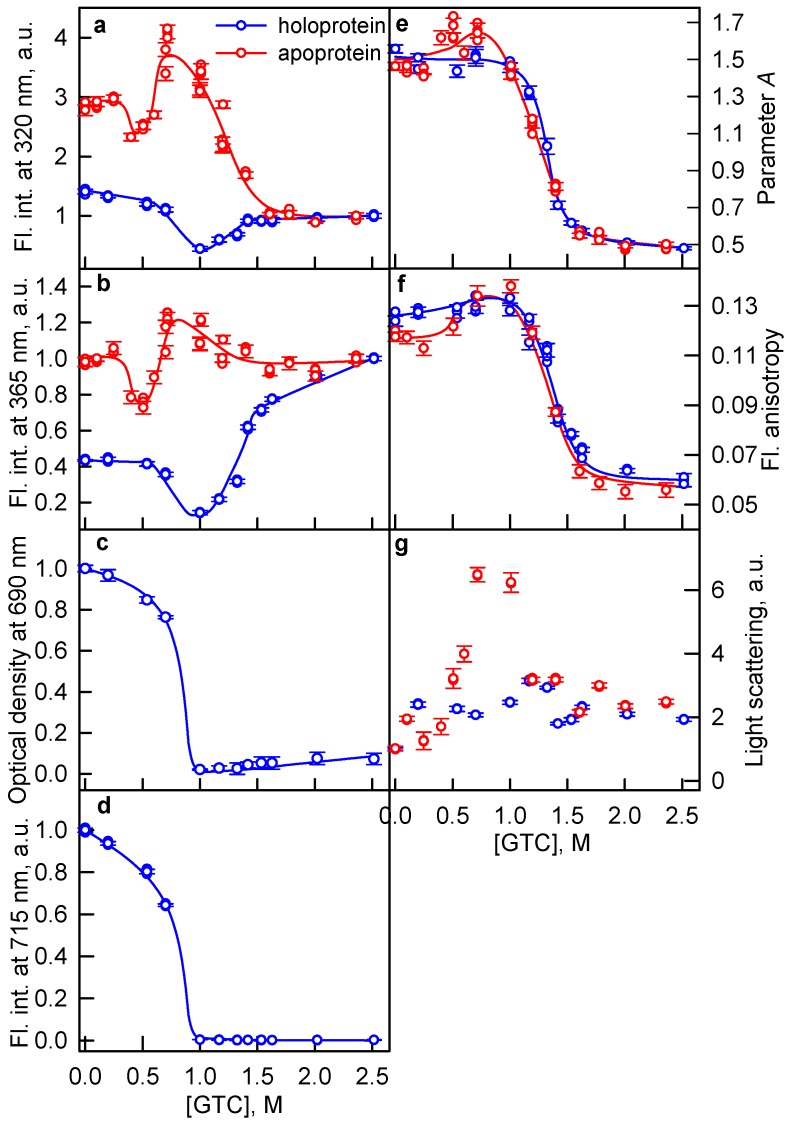
Unfolding of iRFP713 in the apoform and holoform induced by guanidine thiocyanate (GTC). (**a**,**b**) Changes in the tryptophan fluorescence intensity at an excitation wavelength of 295 nm and registration wavelengths of 320 and 365 nm. The values of fluorescence intensity *I*_320_ and *I*_365_ were normalized to unity at high denaturant concentration (2.5 M GTC). (**c**) Changes in optical density of the solution at a registration wavelength of 690 nm. (**d**) Changes in the chromophore fluorescence intensity at an excitation wavelength of 690 nm, corrected for the absorbance of the solution at the excitation wavelength (see Materials and Methods). (**e**) Changes in the parameter *A* = *I*_320_/*I*_365_ at an excitation wavelength of 295 nm. (**f**) Changes in fluorescence anisotropy at an emission wavelength of 365 nm and excitation wavelength of 295 nm. (**g**) Changes in the light scattering. The values of the recorded characteristics for iRFP713 in the apoform and holoform are represented by red and blue circles, respectively. The measurements were performed after 24 h incubation of the native protein in the presence of GTC. Error bars are s.e.m., *n* = 3.

**Figure 2:**
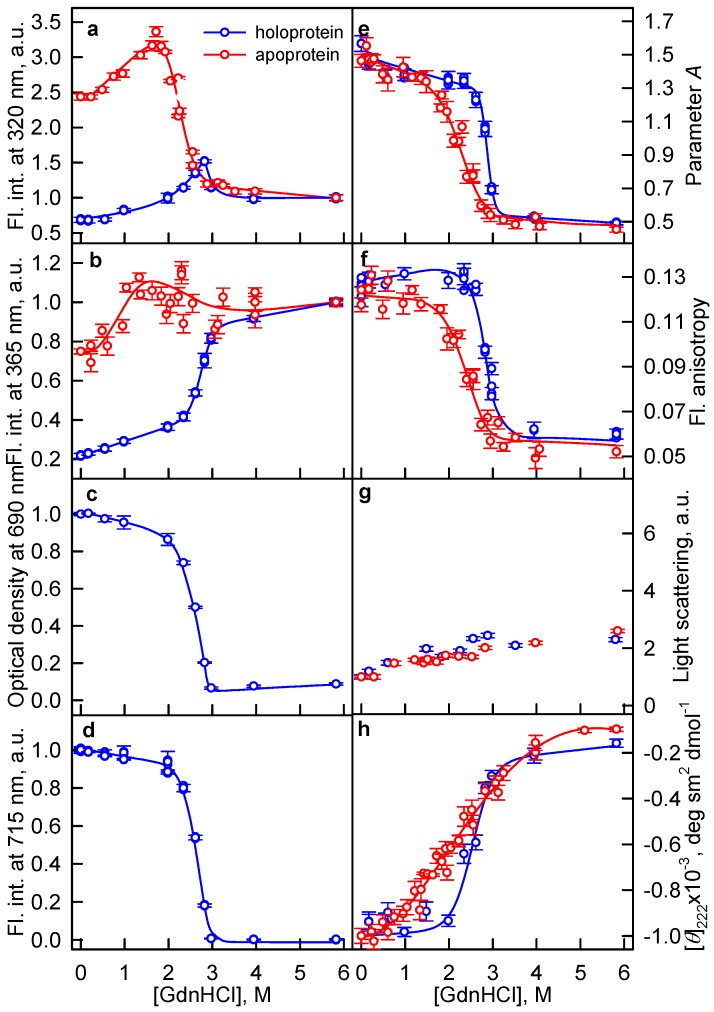
Unfolding of iRFP713 in the apoform and holoform induced by guanidine hydrochloride (GdnHCl). Panels **a**–**g** are the same as in Figure 1. (**h**) Changes in the ellipticity at 222 nm. Symbols as in Figure 1.

The tryptophan fluorescence intensity of iRFP713 in the holoform in the native state is significantly lower than for the protein in the apoform (Figure 1a,b and Figure 2a,b). As was shown previously, quenching of tryptophan fluorescence of iRFP713 in the holoform is caused by nonradiative energy transfer from tryptophan residues of the protein to its chromophore [12].

In the range of GTC concentrations from 0.5 to 1.0 M, the intensity of tryptophan fluorescence of the iRFP713 in the holoform decreased substantially (Figure 1a,b, blue lines). In this range of denaturant concentrations, the optical density and the fluorescence intensity of BV of iRFP713 in the holoform dropped to zero (Figure 1с,d). The latter clearly indicates that, in this range of denaturant concentrations, the protein aggregates and precipitates. The holoprotein iRFP713 retained the values of the parameter *A* and the fluorescence anisotropy close to those for the protein in the native state within this range of GTC concentrations (Figure 1e,f, blue lines). This indicated that the characteristics of tryptophan fluorescence of the portion of the protein remaining in the solution are the same as those for the protein in the absence of the denaturant. The decrease in the tryptophan fluorescence intensity (*I*_320_ and *I*_365_) at the GTC-induced unfolding of the iRFP713 apoform testified that apoprotein starts to aggregate and precipitate at the GTC concentration of 0.4 M (Figure 1a,b, red lines). The fact that the aggregation of iRFP713 in the holoform occurs at higher concentrations of the denaturant is obviously related to the stabilization of the holoprotein by BV incorporated in the pocket of the GAF domain.

In the range of GTC concentrations from 0.5 to 0.7 M, a rise in the tryptophan fluorescence intensity *I*_320_ and *I*_365_ of iRFP713 in the apoform was observed (Figure 1, red lines). We assume that under these experimental conditions, decomposition/dissolution of apoprotein aggregates takes place. In the same range of GTC concentrations, the values of the parameter *A*, the fluorescence anisotropy, and the light scattering intensity of iRFP713 in the apoform slightly increase (Figure 1, red lines). The values of characteristics of iRFP713 in the apoform measured under these conditions can be affected by the presence of small oligomers/aggregates in the solutions of apoprotein. It is well known that globular proteins in the intermediate state, bearing “sticky” hydrophobic clusters on its surface, are capable of aggregation when their surface charge is neutralized [35,36,37]. It is supposed that the neutralization of the surface charge of the protein in the intermediate state is caused by binding to the protein of GdnH^+^ ions in the composition of denaturant used to unfold the protein. It was shown that GdnH^+^ ions can be involved in hydrogen bond formation with the carboxyl groups of glutamic and aspartic acids and with amide groups of glutamines and asparagines [38,39,40]. The surface of iRFP713 is negatively charged at neutral pH (pI = 5.86), meaning that the number of negatively charged carboxyl groups of amino acid side chains on the surface of the protein exceeds the number of positively charged amino groups. The formation of the intermediate state of iRFP713 in the apo- and holoform at a concentration of GTC above 0.4 and 0.5 M, respectively, would explain the aggregation of the protein under these experimental conditions.

At a concentration of GTC from 1.0 to 1.5 M, the tryptophan fluorescence intensity of the iRFP713 holoform increased (Figure 1a,b, blue lines). A minor but clearly distinguishable growth of the optical density of BV was also detected (Figure 1c), the absorption spectra in the visible region of the spectrum under these experimental conditions is typical of that for free BV (the figure is not present). This indicated an increase in the portion of dissolved molecules of the denatured protein in this range of the denaturant concentrations. The unfolding of iRFP713 in the holoform occurred in the range of 1.1–1.7 M GTC and was accompanied by a decrease in the values of the parameter *A* and fluorescence anisotropy (Figure 1e,f, blue lines). The values of the parameter *A* and fluorescence anisotropy of iRFP713 in the holoform nearly coincided in the denaturing transition region with the values of those characteristics of the apoprotein (Figure 1e,f). As in the range of GTC concentrations where the holoprotein unfolds, BV is no longer embedded in the protein structure (Figure 1d), so the holoprotein and the apoprotein behave identically under these conditions. 

**Figure 3:**
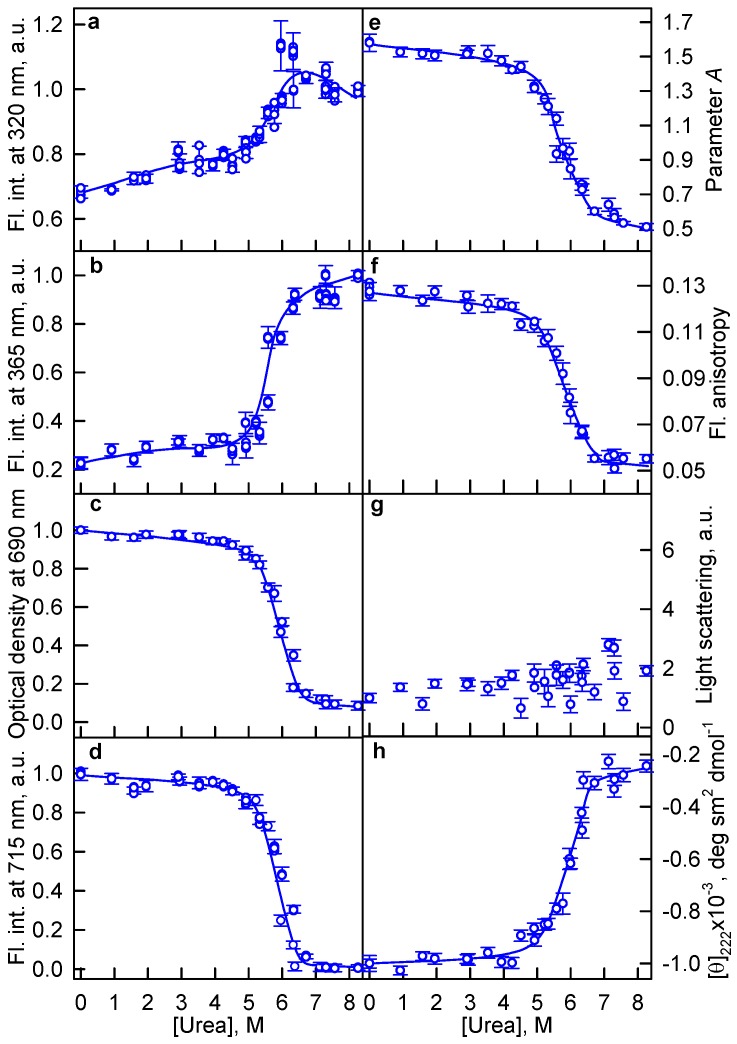
Unfolding of iRFP713 in the holoform induced by urea. See Figure 2 for the details.

There were no local minima detected, indicating a precipitation of aggregates in the case of GTC-induced protein denaturation, on the curves of the tryptophan fluorescence intensity recorded at the unfolding of the iRFP713 holo- and apoprotein in the presence of GdnHCl (Figure 2a,b) and urea (Figure 3a,b). Thus, there are no conditions favoring protein aggregation in the presence of GdnHCl and urea. The absence of aggregation at the urea-induced unfolding of iRFP713 is obviously related to the non-ionic nature of the denaturant. It is known that guanidine is a strong base (pKa = 13.6) [41]. As a result, the salts of guanidine, including GdnHCl and GTC, dissociate into the GdnH^+^ cations and the corresponding counter ions under physiological conditions [42]. Thus, the same concentration of these denaturants in the solution should yield an equal number of GdnH^+^ cations. As GdnHCl is a weaker denaturant compared to GTC, an intermediate state prone to aggregation is formed at higher concentrations of this denaturant. Under these conditions, the increased number of GdnH^+^ groups bound to iRFP713 changes its surface charge to positive which does not facilitate the aggregation.

At the pre-denaturing concentrations of GdnHCl from 0.2 to 1.7 M, a noticeable increase in the tryptophan fluorescence intensity of iRFP713 in the apoform compared to the protein in the absence of denaturant was recorded (Figure 2a,b, red lines), which was not accompanied by a significant change of the fluorescence spectrum of the protein (Figure 2e, red line). Similar changes were observed for iRFP713 in the holoform at the GdnHCl concentration from 0.5 to 2.8 M (Figure 2a,b, blue lines) and at the urea concentration from 1 to 5 М (Figure 3a,b). Notably, the increase in the quantum yield of tryptophan fluorescence of the holoprotein was much less than that found for the apoprotein, probably due to quenching of intrinsic fluorescence of the holoprotein by the chromophore. We suppose that these changes are associated with the formation of an intermediate state of iRFP713, in which tryptophan fluorescence is less quenched than in the native protein.

The GdnHCl-induced unfolding of iRFP713 in the holoform started at higher denaturant concentrations and was more cooperative than the apoprotein unfolding. This indicates that BV resided in the GAF pocket of the holoprotein and stabilized it up to the GdnHCl concentrations at which protein structure disrupts. 

**Figure 4:**
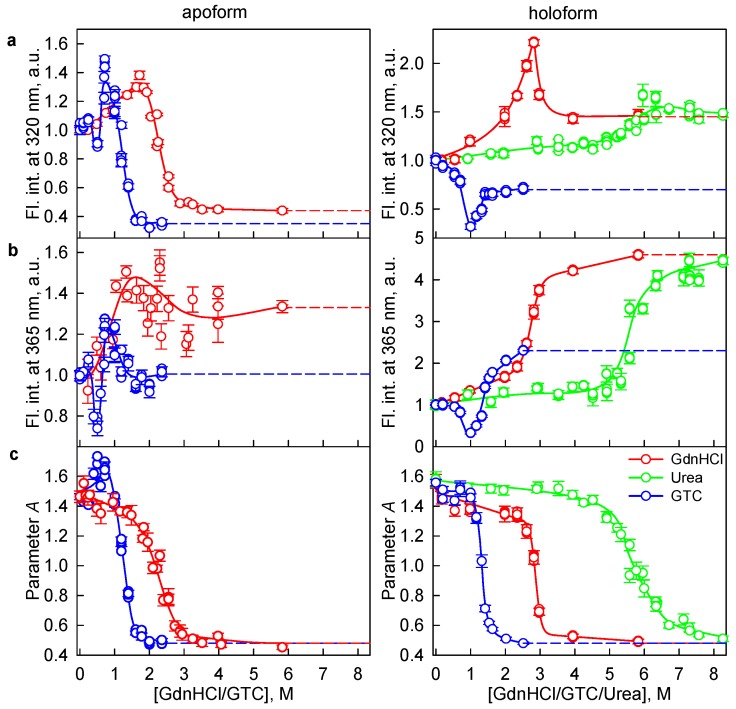
Unfolding of iRFP713 in the apoform (left panels) and holoform (right panels) induced by different chemical denaturants. (**a**,**b**) Changes in the tryptophan fluorescence intensity at an excitation wavelength of 295 nm and registration wavelengths of 320 and 365 nm. The values of fluorescence intensity *I*_320_ and *I*_365_ were normalized to unity at zero concentration of denaturants. (**c**) Changes in the parameter *A* = *I*_320_/*I*_365_ at an excitation wavelength of 295 nm. The values of the recorded characteristics for iRFP713 unfolding induced by GdnHCl, GTC and urea are represented by red, blue, and green circles, respectively. The measurements were performed after 24 h incubation of the native protein in the presence of denaturants. Error bars are s.e.m., *n* = 3.

The parameter *A* values of iRFP713 in the apo- and holoform unfolded in concentrated solutions of GTC (2.5 M, Figure 1e) and GdnHCl (6 M, Figure 2e) were approximately the same and was equal to 0.48 ± 0.01. The iRFP713 holoprotein unfolded in concentrated solutions of urea (8 M) had a slightly higher parameter *A*, reaching a value of 0.51 ± 0.01 (Figure 3e). The dependencies of the tryptophan fluorescence intensity of iRFP713 in the apo- and holoform on the denaturant concentration shown in Figure 1, Figure 2 and Figure 3 were normalized to unity at a high concentration of denaturants. This representation of the data is quite relevant, since the BV molecule covalently attached to the iRFP713 holoform in the unfolded state hardly affects the fluorescence of tryptophan residues. At the same time, the presence of BV molecule in the native state of the iRFP713 holoprotein leads to the quenching of tryptophan fluorescence by nonradiative energy transfer from tryptophan residues to BV chromophore (see above). To analyze the influence of the used denaturants at high concentration on the fluorescence of tryptophan residues of iRFP713 in unfolded state, we normalized the intensity of tryptophan fluorescence to unity at zero concentration of the denaturants (Figure 4). It was found that the tryptophan fluorescence intensities of the iRFP713 holoprotein in concentrated solutions of GdnHCl and urea differed slightly. The values of the tryptophan fluorescence intensity of the iRFP713 apo- and holoprotein, unfolded in GTC and GdnHCl at a high concentration, differed substantially, despite the equality of the parameter *A* under these conditions (Figure 4). A quenching of tryptophan fluorescence by thiocyanate ions (SCN^−^), which are GdnH^+^ counter ions in GTC, can contribute to the observed difference of the tryptophan fluorescence intensity of iRFP713 in concentrated solutions of GdnHCl and GTC. It is believed that urea and GdnH^+^ ions of GdnHCl and GTC denature proteins by preferentially interacting with the groups of polypeptide backbone and of side chains of proteins [39,43]. The SCN^−^ ion of GTC can also engage in hydrogen bonds with amide groups of the protein backbone [44]. The examination of water shell of SCN^−^ and GdnH^+^ ions by neutron scattering revealed that both ions are weakly hydrated. Based on this, it was proposed that SCN^−^ and GdnH^+^ ions can bind to side chains of hydrophobic and, in particular, aromatic amino acids of the protein [44]. In the case of GdnH^+^ ions, this assumption was also supported by the observation that in proteins the guanidinium group of the arginine residue is often located almost parallel to the aromatic rings of the tryptophan and tyrosine side chains [40]. Later, the possibility of a specific interaction of GdnH^+^ cations with aromatic amino acids was disproved [45]. The experimental evidence of the specific interaction of SCN^–^ ions with aromatic amino acids is not yet available. SCN^−^ ions localized in the vicinity of the tryptophan residues of unfolded protein because of binding to protein side chains or to polypeptide backbone may quench the tryptophan fluorescence statically. However, we did not find any decrease in the fluorescence quantum yield of N-acetyl-l-tryptophanamide (NATA) in the presence of NaSCN at high concentration (Supplementary Figure S2). At a high concentration of GdnHCl, an increase in the fluorescence of the fluorophore was detected. Similar changes of the fluorescence intensity of NATA were observed in the presence of NaCl. This argues for the change of spectral properties of NATA in the presence of GdnHCl being caused by a change in the ionic strength of the solution. This also does not exclude that SCN^−^ ions may exert a quenching action on the NATA fluorescence. It is conceivable that a decrease in the fluorescence quantum yield of NATA in the presence of NaSCN due to quenching is compensated by an increase in the fluorescence quantum yield of the fluorophore due to an increase in the ionic strength of the solution under these conditions. At the same time, the fluorescence intensities of NATA at high concentrations of NaSCN and GdnHCl differed less drastically compared to the tryptophan fluorescence intensity of unfolded iRFP713 in concentrated solutions of GTC and GdnHCl. This means that the spectral characteristics of iRFP713 in the fully unfolded state are affected not only by the properties of the solvent. The value of the parameter *A* measured for the tryptophan solution was equal to 0.45 ± 0.01, which is less than the value of this characteristic of iRFP713 under strictly denaturing conditions created by high concentrations of the denaturants used here. This allowed us to conclude that the fully unfolded state of iRFP713 retains a residual structure, while the conformation of the polypeptide chain of the unfolded protein in different denaturants is not identical. The presence of a residual level of the conformational order was repeatedly demonstrated for globular proteins unfolded by chemical denaturants at high concentration [46,47]. Recent studies revealed that unfolded proteins exhibit the local clustering of hydrophobic residues [34].

**Figure 5:**
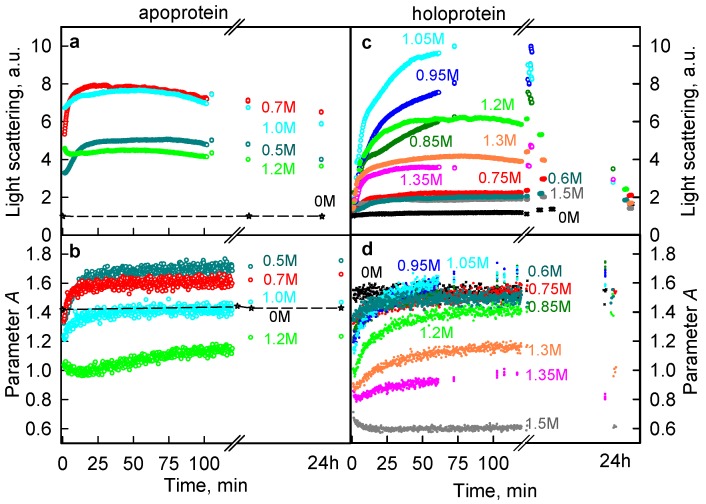
Kinetic traces of GTC-induced unfolding of iRFP713 in the apoform and holoform. (**a**,**c**) Changes in the light scattering of the apoform and holoform, respectively. (**b**,**d**) Changes in the parameter *A* = *I*_320_/*I*_365_ of the apoform and holoform, respectively. The excitation wavelength is 295 nm. Numerals at the curves specify applied GTC concentration.

Analysis of kinetic dependences of the various spectral characteristics of iRFP713 confirmed the formation of aggregates at protein unfolding in the presence of GTC. The aggregation of the iRFP713 apo- and holoprotein was accompanied by drastic growth of the light scattering of the protein with the maximal 8-and 10-fold increase detected at the presence of 0.7 M (Figure 5a) and 1.05 M GTC (Figure 5c), respectively. The protein aggregation was also manifested by a significant increase in the parameter *A* to the value exceeding the level of the native protein (Figure 5b,d). The prolonged incubation of the iRFP713 apo- and holoprotein in a solution of the denaturant resulted in a monotonous decrease of the light scattering after the initial fast rise of the characteristic, which was obviously caused by the precipitation of protein aggregates. This allows us to assume that formed aggregates of iRFP713 have a large size.

### 2.2. Formation of the Monomeric State at Unfolding of iRFP713 in the Holoform

iRFP713 contains three tryptophan residues, W109, W281, and W311, which are located in different protein domains (Supplementary Figure S1). The residues W109 and W281 are positioned in the internal region of the PAS domain and at the periphery of the GAF domain of the protein, respectively. Analysis of the microenvironment of these tryptophan residues allows considering it rather dense and rigid [23]. The W311 residue is located in dimeric interface of iRFP713. We used the advantage of such localization of tryptophan residues in the structure of iRFP713 to identify the protein regions that undergo structural changes at different stages of protein unfolding. To this aim, we generated iRFP713 mutant variants containing single tryptophan residue (two other tryptophan residues were replaced by phenylalanine). These mutant proteins are designated as follows: iRFP713-W109 (iRFP713/W281F/W311F), iRFP713-W281 (iRFP713/W109F/W311F), and iRFP713-W311 (iRFP713/W109F/W281F).

**Figure 6:**
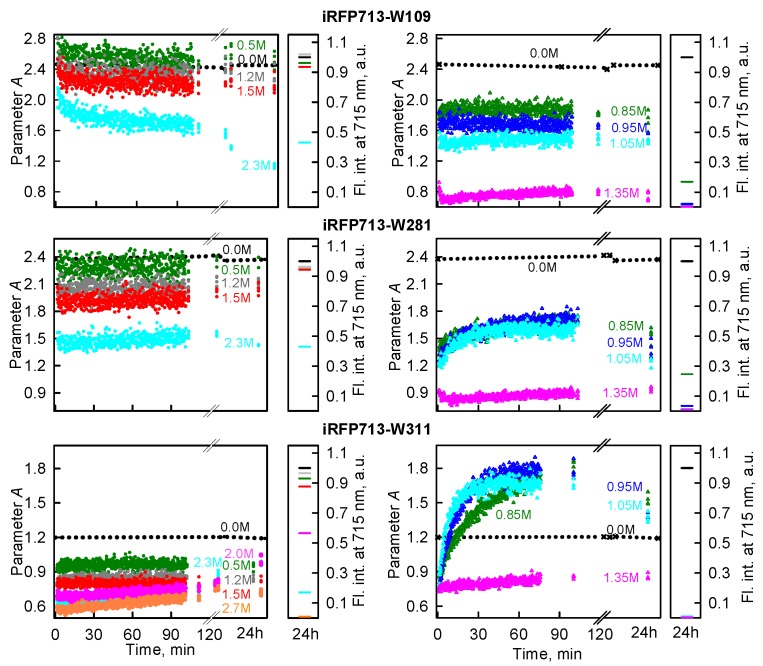
Kinetic traces of unfolding of mutant variants **iRFP713-W109**, **iRFP713-W281**, and **iRFP713-W311** in the holoform induced by GdnHCl (left panel) and GTC (right panel). Changes in the parameter *A* = *I*_320_/*I*_365_ at an excitation wavelength of 295 nm. The values of the chromophore fluorescence intensity at an excitation wavelength of 690 nm after 24 h after mixing the solutions of the mutant protein and denaturant are designated as lines on the right on the panels. Numerals at the curves specify applied denaturant concentration.

Analysis of kinetic dependences of spectral characteristics of a single tryptophan variant of iRFP713 at the GdnHCl- and GTC-induced unfolding (Figure 6) revealed that structural changes in the early stages of the protein unfolding can be mapped mainly to the dimeric interface of iRFP713. The value of the parameter *A* of the mutant variant iRFP713-W311 dropped sharply at the mixing of protein and denaturant to the value typical for proteins in the unfolded state with tryptophan residues highly accessible to the solvent (Figure 6). No further noticeable increases in the parameter *A* of iRFP713-W311 were seen in the presence of GdnHCl at pre-denaturing concentrations (less than 2 M), in contrast to the significant growth of the parameter *A* of iRFP713-W311 in the presence of GTC caused by the protein aggregation. Parameter *A* of the mutant proteins iRFP713-W109 and iRFP713-W281 did not alter significantly immediately after the mixing proteins with GdnHCl in the pre-denaturing concentration (up to 2 M; Figure 6). In the presence of small concentrations of GdnHCl, the mutant variants iRFP713-W109, iRFP713-W281, and iRFP713-W311 retained the BV fluorescence, inherent to these proteins in their native state (Figure 6, lines on the right on the panels). Together, these data allowed us to conclude that the dissociation of the iRFP713 native dimer into monomers occurs early at protein unfolding. 

**Figure 7:**
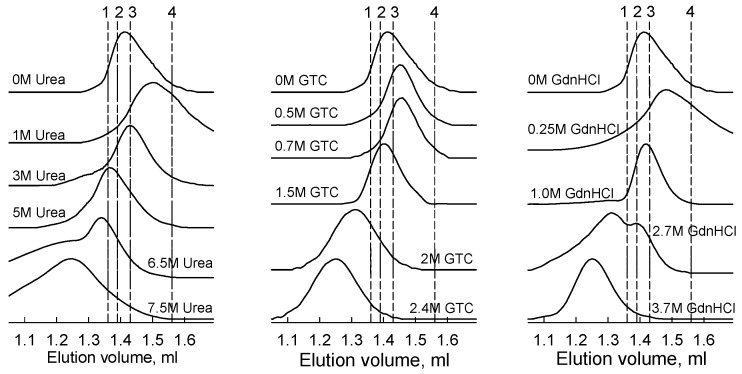
Changes in hydrodynamic dimensions at holoform iRFP713 unfolding induced by urea (**left panels**), by GTC (**central panels**), and by GdnHCl (**right panels**). Changes in elution profile of iRFP713 in the holoform at increasing denaturant concentration. Numerals at the curves specify applied denaturant concentration. Dotted lines designate the positions of the elution peaks of proteins with a known molecular mass used for calibration of the column: 1–158 kDa (aldolase); 2–75 kDa (conalbumin); 3–44 kDa (ovalbumin); 4–29 kDa (carbonic anhydrase).

The dissociation of iRFP713 dimer resulted in theformation of compact monomers of that retained the structure around the tryptophan residues W109 and W281, and BV were intact. We examined the change in spatial structure of iRFP713 in the holoform at GTC-, GdnHCl-, and urea-induced unfolding by gel filtration (Figure 7). The elution peak of iRFP713 in the holoform in the presence of small concentrations of all denaturants actually shifted to higher elution volumes relative to the elution profile of the holoprotein in its native state (Figure 7). These data are in line with the formation of a compact monomer of iRFP713.

At the GdnHCl concentration from 0.5 to 2.5 M and at the urea concentrations from 3 to 5 M, an inverse shift of the elution peak of iRFP713 in the holoform to smaller elution volumes was observed, which can be connected with the formation of a state with a larger hydrodynamic radius under these conditions (Figure 7). A second elution peak detected on the elution profiles of the iRFP713 holoform at high concentrations of GdnHCl and urea was evidently due to the formation of a fully unfolded protein state. The elution profiles of the holoform of iRFP713 pre-incubated in the presence of 1.0–1.2 М GTC contained no elution peaks. The single elution peak recorded for the iRFP713 holoform pre-incubated in the presence of 1.5 M GTC had a significantly smaller area than that of the native protein and was shifted to smaller elution volumes compared to elution profiles of the protein in the presence of 0.5–0.7 М GTC (Supplementary Figure S3). A further increase in the GTC concentration resulted in the appearance of an elution peak on iRFP713 elution profiles typical of a fully unfolded protein, and an increase in its area. Note that the gel-filtration experiments were performed at a higher concentration of iRFP713 in the holoform (*OD*_280_ = 0.3) compared to the protein concentration used in the spectral measurements (*OD*_280_ = 0.06–0.1). The gel-filtration iRFP713 samples with increased protein concentration containing 1.0–1.2 M GTC was clearly opalescent. These data are consistent with the large size of the GTC-induced iRFP713 aggregates, which should elute with a dead volume of the column. Thus, we can assume that iRFP713 molecules of a larger hydrodynamic radius revealed in the presence of 0.5–2.5 M GdnHCl, 3–5 M urea, and probably 1.5 M GTC by the gel filtration method could be oligomers of iRFP713-precursors of large aggregates formed in the presence of GTC. The iRFP713 oligomerization would explain the increase in the parameter *A* to the level of the native dimer observed at the unfolding of the iRFP713 holoprotein in the presence of GdnHCl at a concentration below 2 M, which was evidently connected with the re-shielding of the tryptophan residue W311 from the solvent. The formation of oligomers also correlates with the lack of a rise in the light scattering and the fluorescence anisotropy of tryptophan residues (Supplementary Figure S4a) at the GdnHCl-induced unfolding of iRFP713 in the holoform. The formation of large-sized iRFP713 aggregates in the presence of GTC resulted in a significant increase in the fluorescence anisotropy of holoprotein (Supplementary Figure S4b).

### 2.3. Reasons of the Aggregation of iRFP713 in the Apo- and Holoform

**Figure 8:**
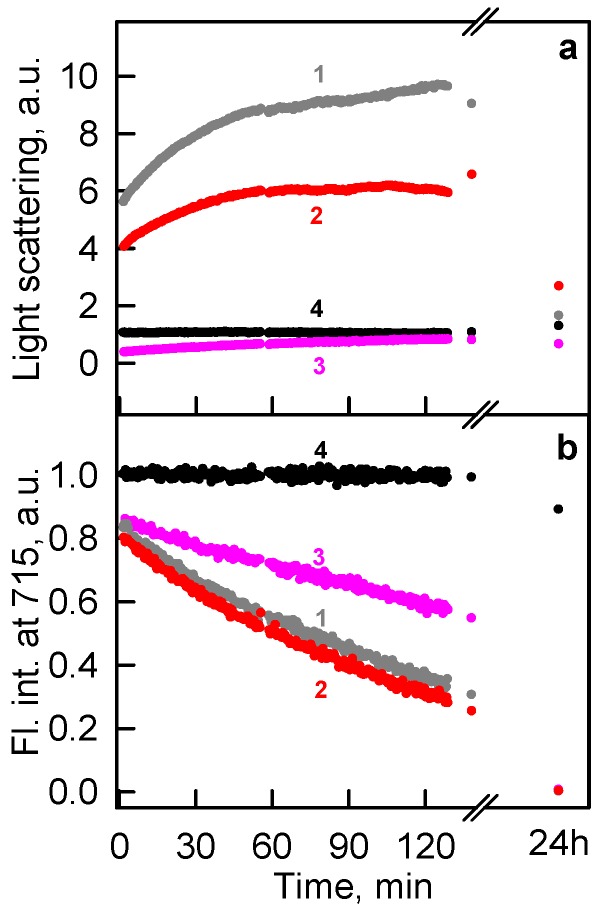
Effect of ATP on the kinetics of GTC-induced unfolding of iRFP713 in the holoform. (**a**) Changes in the light scattering. (**b**) Changes in the parameter *A* = *I*_320_/*I*_365_ at an excitation wavelength of 295 nm. Curves 1, 2, and 3: unfolding induced by 1.0 M GTC in the presence of 0, 10, and 100 mM Mg-ATP; Curve 4: control, 0 M GTC, 0 mM Mg-ATP.

We have revealed that moderate GTC concentrations stimulate a strong aggregation of iRFP713 in the apo- and holoform at the protein unfolding in contrast to GdnHCl or urea. The protein aggregation is probably mediated by the specific interaction of the denaturant with the protein in the intermediate state. To confirm that iRFP713 in the intermediate state has hydrophobic areas on its surface, we conducted an additional experiment. ATP was shown to exhibit hydrotropic properties [48], i.e., it is able to prevent the aggregation of hydrophobic molecules. A decrease in the light scattering of iRFP713 in the holoform at GTC-induced unfolding in the presence of ATP argues for the suppression of aggregation (Figure 8). These data indicated that hydrophobic interactions are involved in aggregation of iRFP713 molecules.

A number of studies have shown that the SCN^−^ ion can form intra- and intermolecular cross-linking accepting two hydrogen bonds [49,50]. Therefore, we decided to test if iRFP713 in the intermediate state interacts only with guanidinium cations or with both ions of GTC. We used heating to stimulate the transition of iRFP713 to the intermediate state. We performed thermal denaturation of iRFP713 in a solution containing NaSCN or GdnHCl at a concentration of 0.015 M. This NaSCN/GdnHCl concentration is insufficient to induce structural changes in the protein, but it provides a significant excess of the SCN^−^/GdnH^+^ anions over the protein molecules (by 5000 times). The dependences of the light scattering of iRFP713 practically coincided for the protein in the absence and in the presence of NaSCN (Supplementary Figure S5). Aggregation of iRFP713 upon thermal denaturation was markedly enhanced in the presence of 0.015 M GdnHCl. The heating of iRFP713 in the presence of GdnHCl resulted in an earlier and more pronounced growth of light scattering in contrast to heating of the protein in the absence or presence of NaSCN. This finding is consistent with preferential binding of GdnH^+^ ions rather than of SCN^−^ ions to iRFP713 in the intermediate state.

## 3. Materials and Methods

### 3.1. Plasmids, Mutagenesis, Protein Expression, and Purification 

The iRFP713 genes were amplified and cloned into a pBAD/His-B vector (Invitrogen, Carlsbad, CA, USA) using BglII and EcoRI sites. LMG194 host cells (Invitrogen, Carlsbad, CA, USA) were co-transformed by pWA23h plasmid for the expression of heme oxygenase under the rhamnose promoter [51] and pBAD/His-B plasmid-encoding iRFP713 and its variants with polyhistidine tags on the N-termini. Bacterial cells were grown in RM medium supplemented with ampicillin and kanamycin. The expression of heme oxygenase was initiated first by 0.02% rhamnose. After incubation of cell culture for 5 h at 37 °C, the expression of the target protein was induced by 0.002% arabinose followed by the incubation of cell culture for 12 h at 37 °C and for 24 h at 18 °C. Proteins were purified with affinity chromatography on an Ni-NTA agarose column (GE Healthcare, Chicago, IL, USA). The Ni-NTA elution buffer contained 100 mM EDTA instead of imidazole. The elution buffer was exchanged to PBS buffer by dialysis. The final purification was achieved with ion-exchange chromatography on a MonoQ column (GE Healthcare). The apoform of iRFP713 was expressed in LMG194 cells. The overnight LMG194 culture was grown for 2–3 h at 37 °C; protein synthesis was then induced by 0.002% arabinose. The subsequent steps of expression and purification of proteins in apoform were the same as those of proteins in holoform. 

The purity of the proteins was tested by sodium dodecyl sulfate polyacrylamide gel electrophoresis (SDS-PAGE) in a 12% polyacrylamide gels [52]. The protein was concentrated and stored in 20 mM Tris/HCl buffer, 150 mM NaCl, pH 8.0. The absorbance of the protein samples did not exceed 0.1, and the measurements were performed in 20 mM Tris/HCl buffer, pH 8.0, containing 1 mM tris (2-carboxyethyl) phosphine (TCEP, Sigma-Aldrich, St. Louis, MO, USA).

GdnHCl, GTC, urea, NaSCN, TCEP, and N-acetyl-l-tryptophanamide (Sigma, St. Louis, MO, USA) were used without further purification. The concentration of GdnHCl, GTC, and urea in stock solutions was determined on the basis of the refraction coefficient using the Abbe refractometer (LOMO, St. Petersburg, Russia).

### 3.2. Spectrophotometric Experiments

Absorption experiments were performed using a U-3900H spectrophotometer (Hitachi, Tokyo, Japan) with microcells 101.016-QS 5 × 5 mm (Hellma, Jena, Germany) at room temperature. The fluorescence spectra were recorded using a Cary Eclipse spectrofluorometer with 10 × 10 cells (Agilent Technologies, Mulgrave, Australia).

### 3.3. Fluorescence Spectroscopy

The fluorescence experiments were performed using a Cary Eclipse spectrofluorimeter (Agilent, Santa Clara, CA, USA) with FLR cells 10 × 10 × 4 mm with a path length of 10 mm (Starna, Atascadero, CA, USA). The tryptophan fluorescence of the protein was excited at the long-wave absorption spectrum edge (λex = 297 nm) to minimize the contribution of the tyrosine residues in the bulk protein fluorescence. The position and form of the fluorescence spectra were characterized on the basis of parameter *A* = *I*_320_/*I*_365_, where *I*_320_ and *I*_365_ are the fluorescence intensities at the emission wavelengths of 320 and 365 nm, respectively [53]. The value for parameter *A* was corrected for the instrument sensitivity. The specific near-infrared fluorescence of iRFP713 in holoform was excited at 690 nm, and emission was detected at 713 nm. The recorded fluorescence intensity was corrected for the primary inner filter effect according to the approach in [54,55]. The anisotropy of tryptophan fluorescence was calculated using the equation

(1) $$r=\frac{\left(I_{V}^{V}-GI_{H}^{V}\right)}{\left(I_{V}^{V}+2GI_{H}^{V}\right)}\;$$

where $I_{V}^{V}$ and $I_{H}^{V}$ are vertical and horizontal components of the fluorescence intensity excited by vertically polarized light, respectively, and $G=I_{V}^{H}/I_{H}^{H}$ is the coefficient that determines the different instrument sensitivity for the vertical and horizontal components of the fluorescence light, λem = 365 nm [56].

The unfolding of the protein was initiated by manually mixing a 50 μL aliquot of the native protein with 500 μL of a buffer solution containing the desired concentration of denaturant. According to the control experiments, the dead time in these manual mixing-based unfolding-refolding experiments was about 4 s [29,57]. 

The steady-state denaturant-dependent fluorescent characteristics of iRFP713 were recorded after protein incubation in a solution an appropriate denaturant concentration at 23 °С for 24 h. Further increases in the equilibration time did not result in noticeable changes in the detected characteristics.

### 3.4. Gel Filtration Experiments

Gel filtration experiments on iRFP713 unfolded by GTC, GdnHCl, or urea were performed on a Superose 12 PC 3.2/30 column (GE Healthcare) using an AKTApurifier system (GE Healthcare). The samples of iRFP713 were prepared in buffer consisting of 50 mM NaH_2_PO_4_, 150 mM NaCl, pH 8.0 and containing the desired denaturant concentration. Protein samples were pre-incubated at 23 *°*C for 24 h. Ten microliters of the iRFP713 sample were then loaded on the column equilibrated with the same denaturant concentration. A set of proteins with known molecular mass (chromatography standards from GE Healthcare) was used for column calibration.

### 3.5. Circular Dichroism Measurements

Jasco-810 spectropolarimeter (Jasco, Tokyo, Japan) was used for the measurement of circular dichroism (CD) spectra in the far UV range (from 260 to 190 nm). The far-UV CD spectra were recorded in a 1 mm path length cell. Three scans of CD spectra were collected, averaged, and corrected by a buffer solution background for every probe.

## 4. Conclusions

In this study, we showed that the unfolding of iRFP713 in the chromophore-bound and chromophore-free states is accompanied by the dissociation of the native dimer and the subsequent formation of a monomeric intermediate state in which hydrophobic regions are exposed to the protein surface. The monomeric intermediate state of iRFP713 is capable of forming large aggregates when it accumulates at concentrations of GdnH^+^ ions, which provides the neutralization of surface charge of the protein. If the monomeric intermediate state of iRFP713 is populated out of this specific range of GdnH^+^ concentrations, the protein surface would bear either a negative charge that is not compensated by a low number of GdnH^+^ ions bound to the carboxyl groups of glutamic and aspartic acids or a positive charge as a result of GdnH^+^ ions binding to amide groups of glutamines and asparagines. This partially or completely prevents the aggregation of the protein. The aggregation of the protein upon neutralization of the charge on the macromolecule surface can be considered as a hallmark of the formation of the intermediate state of the protein. The approach used in the present work based on unfolding of a protein by a set of denaturants can be applied to detect an intermediate state of a protein with hydrophobic patches on its surface if the traditional methods fail to make this.

## Supplementary Materials

Supplementary materials can be found at http://www.mdpi.com/1422-0067/19/9/2776/s1. Figure S1: The location of tryptophan residues in iRFP713; Figure S2: Changes in spectral properties of N-acetyl-L-tryptophanamide in the presence of GdnHCl, NaSCN, and NaCl; Figure S3: Changes in hydrodynamic dimensions at GTC-induced unfolding of iRFP713 in the holoform; Figure S4: Kinetic traces characterizing the changes in fluorescence anisotropy at the unfolding of iRFP713 in the holoform induced by GdnHCl and GTC; Figure S5: Thermal denaturation of iRFP713 in the holoform.
