# Supplementary material for: The Pathways of the iRFP713 Unfolding Induced by Different Denaturants
Source: Int J Mol Sci. 2018 Sep 15;19(9):2776. doi: 10.3390/ijms19092776 (PMC6163377; doi:10.3390/ijms19092776)
Supplement: Supplementary file 1 [file ijms-19-02776-s001.pdf]

International Journal of
Molecular Sciences

The pathways of the iRFP713 unfolding induced by different denaturants

Olesya V. Stepanenko, Olga V. Stepanenko, I.M. Kuznetsova, K.K. Turoverov

Supplementary Figures

Supplementary Figure S1. The location of tryptophan residues in iRFP713.

Supplementary Figure S2. Changes of spectral characteristics of N-acetyl-L-tryptophanamide in the presence of GdnHCl, NaSCN and NaCl.

Supplementary Figure S3. Changes of hydrodynamic dimensions at GTC-induced unfolding of iRFP713 in the holoform.

Supplementary Figure S4. Kinetic traces characterizing the changes of fluorescence anisotropy at unfolding of iRFP713 in the holoform induced by GdnHCl and GTC.

Supplementary Figure S5. Thermal denaturation of iRFP713 in the holoform.

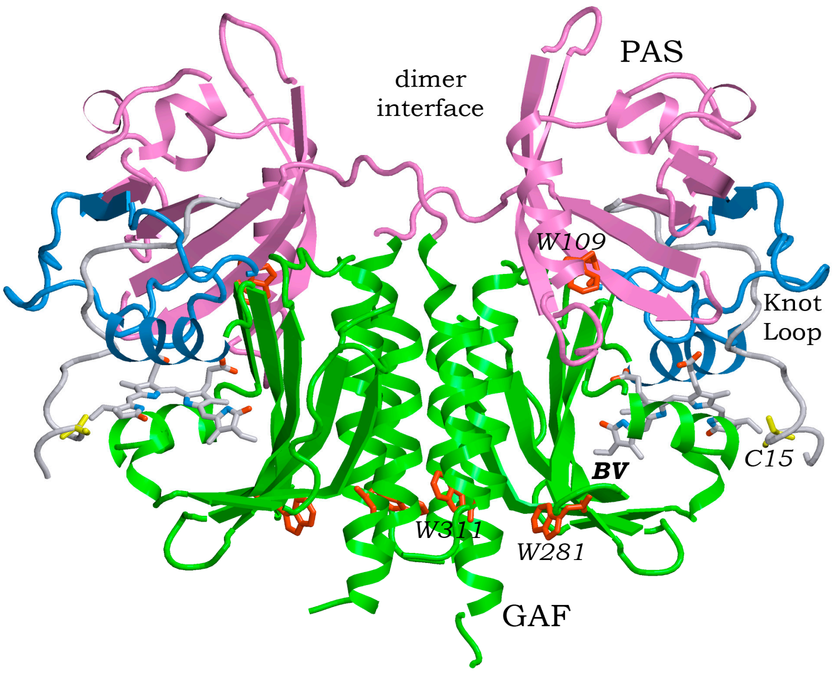

Figure S1. The location of tryptophan residues in iRFP713. The Trp residues are shown in red sticks. The location of the BV chromophore and Cys15 is also shown. PAS and GAF domains of iRFP713 are marked in pink and green, respectively. N-terminal extension and knot-forming loop of PAS domain are indicated in gray and blue. The drawing was made on the basis of the X-ray data of the CBD of the bacterial phytochrome CBD of bacterial phytochrome RpBphP2 (4E04.ent file) [1] deposited in PDB [2] using the graphic software VMD [3] and Raster3D [4].

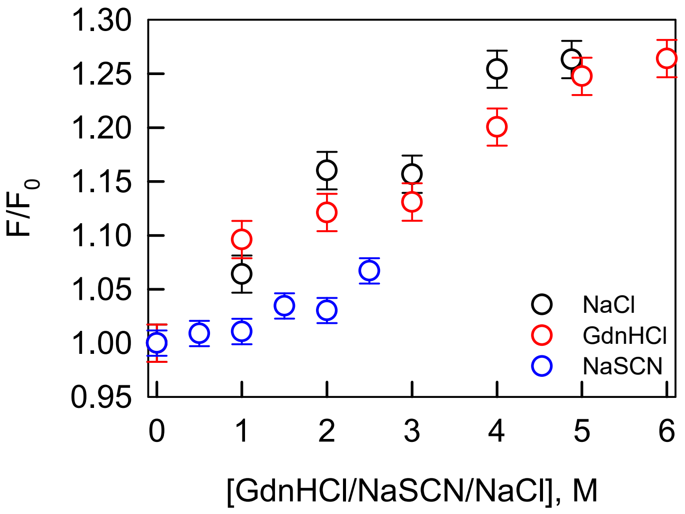

Figure S2. Changes of spectral properties of N-acetyl-L-tryptophanamide in the presence of GdnHCl, NaSCN and NaCl. Changes of total fluorescence of NATA (from 300 to 450 nm). An excitation wavelength is 295 nm. The data are corrected for the primary inner filter effect accounting for the absorbance of the solution at the excitation wavelength [5]. The total fluorescence of the fluorophore in the absence of salts is put to be equal to unity. Different color dots denote different conditions: protein in solutions containing NaCl (black), GdnHCl (red) or NaSCN (blue). Error bars are s.e.m., n=3.

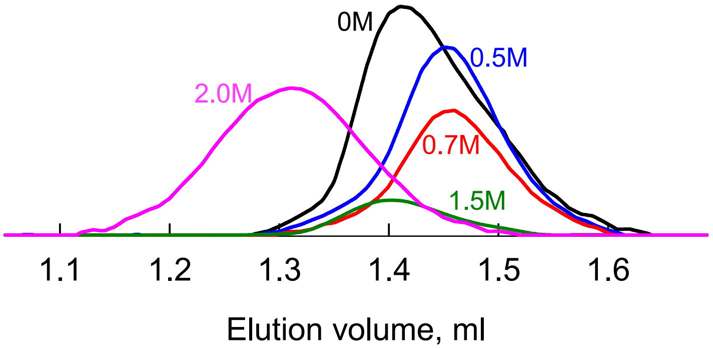

Figure S3. Changes of hydrodynamic dimensions at GTC-induced unfolding of iRFP713 in the holoform. Changes of elution profile of iRFP713 in the holoform at increasing denaturant concentration. Numerals at the curves specify the final GTC concentration.

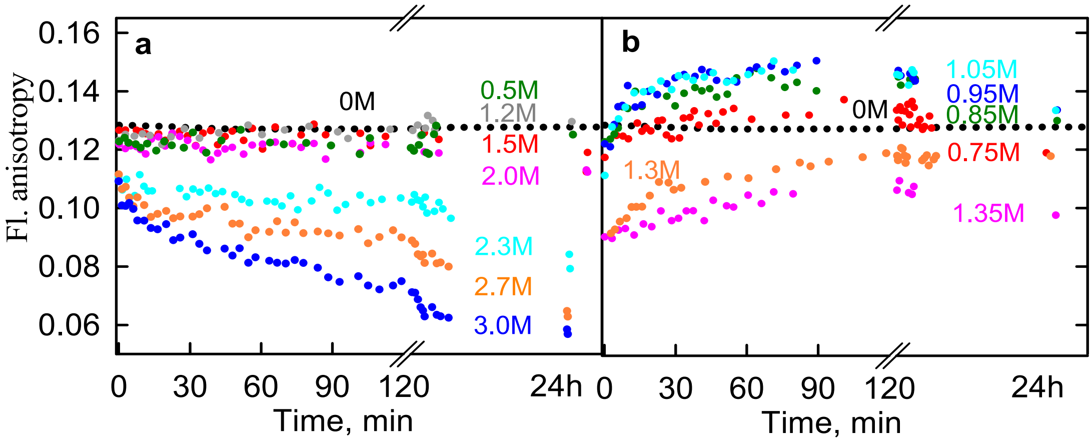

Figure S4. Kinetic traces characterizing the changes of fluorescence anisotropy at unfolding of iRFP713 in the holoform induced by GdnHCl (*a*) and GTC (*b*). An excitation and emission wavelengths are 295 and 365 nm. Numerals at the curves specify the final denaturant concentration in the holoform solutions.

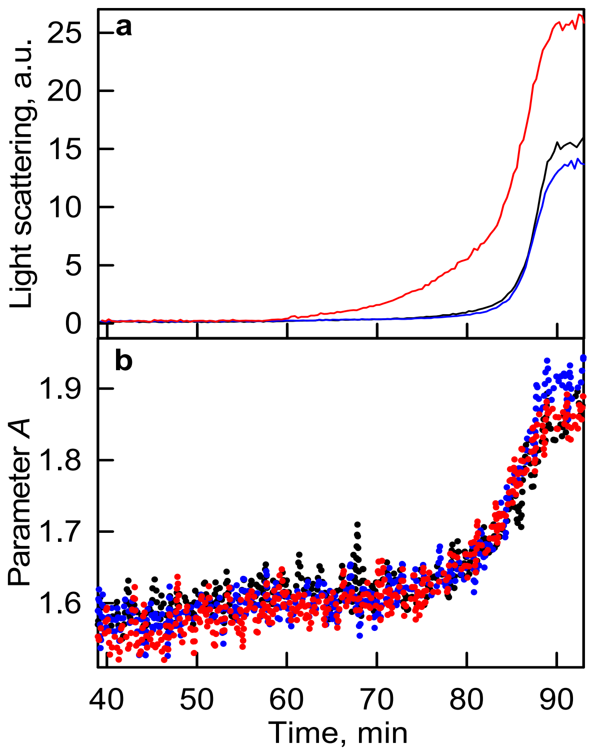

Figure S5. Thermal denaturation of iRFP713 in the holoform. (***a***) Changes in the light scattering. (***b***) Changes in the parameter $A = I_{320}/I_{365}$ at an excitation wavelength of 295 nm. Different color curves denote different conditions: protein in a buffer solution (black), and in solutions containing 0.015 M NaSCN (blue) or GdnHCl (red).

Refferences

1. Bellini D, Papiz MZ (2012) Dimerization properties of the RpBphP2 chromophore-binding domain crystallized by homologue-directed mutagenesis. Acta Crystallogr D Biol Crystallogr 68: 1058-1066.
2. Dutta S, Burkhardt K, Young J, Swaminathan GJ, Matsuura T, et al. (2009) Data deposition and annotation at the worldwide protein data bank. Mol Biotechnol 42: 1-13.
3. Hsin J, Arkhipov A, Yin Y, Stone JE, Schulten K (2008) Using VMD: an introductory tutorial. Curr Protoc Bioinformatics Chapter 5: Unit 5 7.
4. Merritt EA, Bacon DJ (1997) Raster3D: photorealistic molecular graphics. Methods Enzymol 277: 505-524.
5. Fonin AV, Sulatskaya AI, Kuznetsova IM, Turoverov KK (2014) Fluorescence of dyes in solutions with high absorbance. Inner filter effect correction. PLoS One 9: e103878.
